# Does metformin affect ovarian morphology in patients with polycystic ovary syndrome? A retrospective cross-sectional preliminary analysis

**DOI:** 10.1186/1757-2215-2-5

**Published:** 2009-05-31

**Authors:** Angela Falbo, Francesco Orio, Roberta Venturella, Erika Rania, Caterina Materazzo, Achille Tolino, Fulvio Zullo, Stefano Palomba

**Affiliations:** 1 Departments of Obstetrics & Gynecology, University "Magna Graecia" of Catanzaro, Catanzaro, Italy; 2 Endocrinology, University "Parthenope" of Naples, Naples, Italy; 3 University "Federico II" of Naples, Naples, Italy

## Abstract

**Background:**

The significance of polycystic ovarian morphology and its relation to polycystic ovary syndrome (PCOS) is unclear, but probably it is associated with higher androgen and insulin levels and lower sex hormone binding globulin (SHBG) in absence of identifiable differences in gonadotropin dynamics. The aim of this study was to evaluate ovarian morphology in patients affected by PCOS with different ovulatory responses to metformin.

**Methods:**

In this cross-sectional analysis, we studied 20 young normal-weight PCOS patients who had received a six-month course of metformin treatment. Ten of these patients remained anovulatory (anovulatory group), whereas other ten became ovulatory, but failed to conceive (ovulatory group). Other ten age- and body mass index (BMI)-matched PCOS subjects were also enrolled as controls and observed without any treatment (control group).

**Results:**

After six months of metformin, in both PCOS treated groups, a similar improvement in testosterone (T) and insulin resistance indexes was observed. Moreover, in one (10.0%) and nine (90.0%) subjects from anovulatory and ovulatory PCOS groups, respectively, ovarian morphology changed, whereas a significant reduction in ovarian dimension was observed in the PCOS ovulatory group only.

**Conclusion:**

PCOS patients under metformin administration demonstrate a change in ovarian morphology closely related to ovulatory response.

## Background

Polycystic ovary syndrome (PCOS) was firstly defined by the presence of oligo/amenorrhea and hyperandrogenism in association with polycystic ovary (PCO) morphology seen at the time of surgery [1] and, thereafter, observed by ultrasound [2]. Moreover, PCO morphology is not pathognomonic of PCOS because it was also found in childhood, adolescence [3,4], menopausal women [5,6], and in patients with clinical evidence of hyperandrogenism in absence of irregular menstrual cycles [7-9].

The clinical significance of ovarian morphology alone or combined with other PCOS features is still unclear. However, few reports from the previous studies [7,10-13] suggested that this finding is often associated to abnormal gonadotropin levels, lower levels of insulin growth factor-binding protein-1 (IGF-BP1), increased insulin resistance and increased ovarian 17-hydroxiprogesterone (17-OHP) and androgen responses to gonadotropins-releasing hormone (GnRH)-agonists.

Metformin is an insulin sensitizing drug that has been recently introduced for treating women with PCOS due to the knowledge that insulin resistance with compensatory hyperinsulinemia is probably a key factor for the syndrome's pathogenesis [14]. The exact mechanism through metformin acts in PCOS is still unknown. Certainly, metformin exerts systemic actions on glucose-insulin metabolism regulation [15,16], even if a cause-effect relationship between its systemic actions and improved features of PCOS has not been demonstrated yet [16]. In addition, peripheral effects of metformin, dependent and/or independent of its insulin-sensitizing action, have been also found in several experimental studies [16,17]. In particular, our previous data suggested a specific effect of metformin on ovaries, showing that PCOS patients ovulating under treatment had an improved ovarian artery blood flow, and a better dominant follicle and corpus luteum vascularization [17].

To date, there are no studies investigating the relationship between functional response to metformin and ovarian morphological and/or structural changes. Based on these considerations, the aim of the present study was to evaluate metformin effects on ovarian morphology in patients with PCOS who had showed a different response to the treatment.

## Methods

The procedures used were in accordance with the guidelines of the Helsinki Declaration on Human Experimentation and the Good Clinical Practice (CGP) guidelines. No approval by the Institutional Review Board was required due to the retrospective nature of the study. However, a written consent was obtained by all patients for their data processing before beginning the study.

Clinical charts of patients, who referred to our Department for PCOS-related disorders within the last five years, were carefully screened and, among them, 30 young normal-weight PCOS patients were successively enrolled. Diagnosis of PCOS was initially based on the presence of both chronic anovulation and clinical and/or biochemical hyperandrogenism [18]. All PCOS subjects had originally bilateral polycystic ovary (PCO), as defined by previous diagnostic criteria [19].

Twenty PCOS patients had received metformin at the same regimen (daily two tablets 850 mg each) during the previous six months. Ten of these subjects remained anovulatory (anovulatory group) despite treatment, whereas other ten patients became ovulatory but failed to conceive (ovulatory group). Normal ovulatory status was defined by plasma progesterone (P) assay [> 10 ng/mL, (SI: 32 nmol/L)] performed seven days before the expected menses and by the presence of regular menstrual bleedings in three consecutive evaluations.

Other 10 PCOS subjects, who did not receive any treatment and remained anovulatory throughout the following six months, were considered as controls (control group). Ovulatory, anovulatory and control patients were matched for age and body mass index (BMI, kg/m^2^).

Exclusion criteria were considered as: age less than 18 or higher than 35 years, BMI less than 18 or higher than 25, presence of neoplastic, endocrine, metabolic, hepatic and cardiovascular disorders or other concurrent medical illnesses, and current or previous (within the last six months) use of hormonal drugs. In addition, subjects with previous pelvic surgery and organic pelvic diseases, and women intentioned to start a diet or a specific program of physical activity were excluded.

Biochemical, clinical, and ultrasonographic data, performed at baseline and at six-month follow-up were collected.

A complete hormonal and metabolic pattern was recorded for each subject. Free androgen index (FAI) [T (nmol/l)/SHBG × 100], homeostasis model analysis (HOMA) [fasting glucose (mmol/L) × fasting insulin (μU/mL)/22.5] [20] and the fasting glucose-to-insulin ratio (GIR) (mg/10^-4^U) were also calculated.

Anthropometric measurements [including height, weight, BMI and waist-to-hip ratio (WHR)], Ferriman-Gallwey score [21], and ultrasonographic data were noted for each subject. Transvaginal ultrasonographic examinations had been performed by the same experienced operator (A.F.) during the early follicular phase (2^nd^–3^rd ^day) of a spontaneous or P-induced bleedings, and ovarian dimension and morphology were noted bilaterally in each subject. In particular, ovarian dimensions had been obtained by measuring the main three diameters and applying the ellipsoid formula, and ovarian morphology had been defined as PCO or not PCO according to published criteria [19].

### Statistical analysis

The normal distribution of continuous variables was evaluated by using the Kolmogrov-Smirnov test, and continuous data were expressed as mean ± standard deviation (SD). Continuous variables were analyzed with the one-way analysis of variance (ANOVA) and ANOVA for repeated measures with Bonferroni test for the post-hoc analysis.

The Pearson *chi*-square test was performed for categorical variables; conversely, the Fisher's exact test was required for the frequency tables when more than 20% of the expected values were less than 5.

The present study is a retrospective analysis on few PCOS patients for each group. Furthermore a post-study power and the sample size for ovarian morphology change rate were calculated in order to design a well powered (> 80%) RCT. The post-study power analysis and the sample size calculation were performed by the use of SamplePower release 2.0.

Statistical significance was set at *P *< 0.05 for all statistical analyses. The Statistics Package for Social Science (SPSS 14.0.1, 18 Nov 2005; SPSS Inc., Chicago, IL, USA) was used.

## Results and discussion

In our population, both the National Institute of Health (NIH) and the European Society for Human Reproduction (ESHRE)/American Society of Reproductive Medicine (ASRM) [16] for PCOS diagnosis were satisfied.

No difference at baseline was detected in any parameter evaluated among groups (Table 1). After six months of treatment, testosterone (T), androstenedione (A), SHBG and fasting insulin levels, FAI, GIR and HOMA resulted significantly (*P *< 0.05) changed from baseline in both PCOS treated groups (Table 1). At the same time, significant (*P *< 0.05) differences between anovulatory and ovulatory PCOS groups were observed in SHBG, fasting insulin, GIR and HOMA (Table 1). Lastly, the mean variation between anovulatory and ovulatory PCOS groups was not different in the clinical, hormonal and metabolic parameter evaluated (Table 1).

**Table 1:** Clinical, hormonal and metabolic data of PCOS treated patients (anovulatory and ovulatory groups) and PCOS untreated controls (control group) at baseline and at six-month follow-up.

**Group**	**Anovulatory PCOS (n. 10)**		**Ovulatory PCOS (n.10)**		**Control (n. 10)**	
	Baseline	Six months	Baseline	Six months	Baseline	Six months
Age (years)	28.20 ± 3.45	28.20 ± 3.42	28.10 ± 3.31	28.10 ± 3.33	28.40 ± 3.43	28.40 ± 3.43
BMI (Kg/m^2^)	22.92 ± 1.51	23.84 ± 1.46	22.93 ± 1.71	22.81 ± 2.08	22.99 ± 1.71	23.13 ± 1.98
WHR	0.85 ± 0.11	0.83 ± 0.14	0.84 ± 0.13	0.84 ± 0.12	0.86 ± 0.10	0.86 ± 0.16
Ferriman-Gallwey score	12.70 ± 2.41	12.70 ± 2.26	12.13 ± 2.34	11.81 ± 2.48	12.68 ± 2.53	12.54 ± 1.94
FSH (mIU/mL)	5.83 ± 1.40	5.82 ± 1.25	5.78 ± 1.51	5.68 ± 1.52	5.63 ± 1.70	5.62 ± 1.27
LH (mIU/mL)	12.65 ± 3.51	12.14 ± 1.52	11.73 ± 3.64	11.58 ± 3.56	12.90 ± 4.15	12.30 ± 3.00
TSH (μU/mL)	3.10 ± 0.73	3.14 ± 0.49	2.97 ± 0.83	2.99 ± 0.62	3.0 ± 0.68	3.00 ± 0.52
PRL (ng/mL)	9.52 ± 1.81	10.02 ± 1.97	9.12 ± 2.31	8.99 ± 2.23	9.89 ± 2.02	10.09 ± 1.32
E_2_(pg/mL)	48.80 ± 14.95	48.18 ± 14.90	52.28 ± 17.02	53.93 ± 13.39	51.77 ± 9.10	52.55 ± 14.60
P (ng/mL)	1.27 ± 0.45	1.26 ± 0.31	1.39 ± 0.42	1.29 ± 0.62	1.43 ± 0.36	1.46 ± 0.34
17-OHP (μg/L)	1.74 ± 0.50	1.59 ± 0.82	1.54 ± 0.53	1.50 ± 0.63	1.84 ± 0.50	1.93 ± 0.71
T (ng/mL)	4.70 ± 1.23	4.55 ± 1.11*	5.01 ± 1.64	3.41 ± 0.98*	5.15 ± 1.58	5.20 ± 0.78
A (ng/mL)	4.59 ± 1.99	4.34 ± 1.80*	5.16 ± 1.74	3.23 ± 1.07^	4.97 ± 1.36	4.75 ± 0.99
DHEAS (ng/mL)	2690.01 ± 195.67	2653.48 ± 126.05	2685.72 ± 204.65	2557.25 ± 437.86	2511.82 ± 242.16	2483.07 ± 562.54
SHBG (nmol/L)	31.4 ± 1.78	35.90 ± 1.66*°	32.40 ± 3.86	42.82 ± 2.39^	32.10 ± 2.51	33.64 ± 2.45
FAI (%)	15.06 ± 4.35	14.37 ± 4.15*	14.97 ± 4.39	10.44 ± 3.01^	13.60 ± 3.39	12.94 ± 3.19
Fasting glucose (mmol/L)	4.72 ± 0.45	4.79 ± 0.33	4.65 ± 0.50	5.03 ± 0.98	4.73 ± 0.38	4.84 ± 0.43
Fasting insulin (μU/mL)	16.24 ± 3.60	14.94 ± 2.36*°	15.63 ± 4.94	12.98 ± 1.53*	17.92 ± 4.35	12.27 ± 0.84
GIR (mg/10^-4 ^U)	5.59 ± 1.16	6.53 ± 1.00*°	5.96 ± 1.62	7.38 ± 1.14*	5.32 ± 1.39	5.45 ± 0.92
HOMA	3.32 ± 0.61	3.02 ± 0.46*°	3.10 ± 0.62	2.61 ± 0.39*	3.51 ± 0.66	3.47 ± 0.25

* *P *< 0.05 *vs*. baseline; ^ *P *< 0.001 *vs*. baseline; ° *P *< 0.05 *vs*. ovulatory PCOS group.

At enrollment, all PCOS patients had bilateral PCO. After six months of treatment, ovarian morphology changed in one and nine subjects from anovulatory and ovulatory PCOS groups, respectively [1/10 (10.0%) *vs*. 9/10 (90.0%), respectively; *P *< 0.001], while no change was observed in the control group. In particular, only two patients from the ovulatory PCOS group had no PCO morphology, whereas in the others a unilateral PCO morphology was observed.

**Figure 1:**
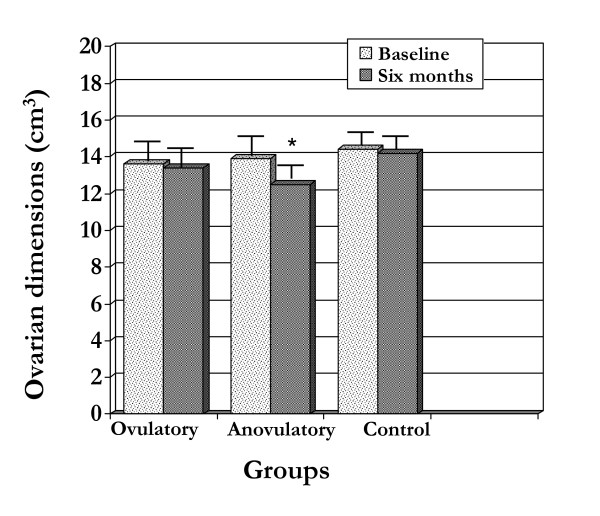
**Ovarian dimension (cm^3 ^± SD) in PCOS patients (anovulatory and ovulatory groups) and controls (control group) at baseline and at six-month follow-up**. * *P *< 0.05 *vs*. baseline.

At baseline, no significant difference was observed among groups in ovarian dimensions (13.9 ± 1.1 *vs*. 13.6 ± 1.0 *vs*. 13.6 ± 1.0 for anovulatory PCOS, ovulatory PCOS and controls, respectively) (Figure 1). In addition, no change from baseline in ovarian dimensions was observed after six months in the anovulatory PCOS group and in controls (13.4 ± 1.0 *vs*. 14.2 ± 1.6, respectively), whereas a significant reduction was observed in the ovulatory PCOS group (13.9 ± 1.1 *vs*. 12.5 ± 2.4; *P *= 0.035).

Considering that the ovarian morphology changed in 90% and 10% of the ovulatory and anovulatory groups, respectively, the post-study power analysis showed a power of > 90% for this study, and very few patients per group will be required in order to detect the effect of metformin on ovarian morphology with a power of 80%.

Our study was aimed to find, if any, a relationship between the systemic effects on hyperinsulinemia and insulin resistance due to the administration of a largely used insulin sensitizing agent, such as metformin, and modification in ovarian morphological features of PCOS patients.

In a recent study [13] on patients affected by PCOS according to the NIH diagnostic criteria, a prevalence of 95% of ovarian dimension and/or structure alterations was found. In addition, the Pearson's correlation analysis showed that the single factor closely related to ovarian volume was the insulin levels, whereas no other significant correlation between altered ovarian morphology and biochemical features of PCOS was observed [13].

On the other hand, a significant higher antral follicles count (AFC) was observed in insulin resistant PCOS patients in comparison with not insulin resistant ones, and a direct relationship between AFC and GIR was successively demonstrated [22].

Considering these findings, the present study analyzed the effects of metformin on ovarian morphology in two populations of young normal-weight PCOS patients who ovulated or did not ovulate under treatment.

As expected, systemic effects of metformin on androgen levels and insulin sensitivity indexes were reported in both ovulatory and anovulatory PCOS patients under treatment.

Even if the meaning of ovarian structure remains debated [23-25], our preliminary results on few patients showed significant change in both ovarian dimension and morphology only in PCOS women who ovulated under metformin. In fact, in 90% of patients who responded to the treatment were reported ovarian morphologic changes. Specifically, in only two out of ten patients PCO morphology disappeared in both ovaries, whereas in the others a unilateral PCO morphology was observed.

Similarly, ovarian volume was significantly reduced after metformin only in patients ovulating after treatment, whereas no significant change was reported in patients who remained anovulatory such as in untreated PCOS controls.

Current results are in agreement with those obtained in a recent randomized controlled study, in which Romualdi *et al*. [27] hypothesized a peripheral effect of metformin independent to its insulin-sensitizing properties. The authors [27] showed an improved clinical and biochemical hyperandrogenism and a reduced ovarian volume and stromal compartment in normal-weight normoinsulinemic PCOS patients after three and six months of metformin, without any effect on glucose and insulin metabolism.

On the other hand, six months of metformin administration was demonstrated to have beneficial effects on follicle growth in women with PCOS, as demonstrated by decrease of anti-Müllerian hormone levels, such as of follicle number and ovarian volume [28]. Furthermore, no hormonal and metabolic data were evaluated after treatment, thus no correlation with ovarian morphologic changes was feasible to find.

Finally, a significant acute effect of one-week metformin administration in PCOS patients was observed in AFC, even if a significant improvement of insulin sensitivity was detected at the same time [22]. Unfortunately, based on these considerations, it is still unclear if the changes in ovarian morphology observed only in patients ovulating under treatment could be considered as a direct effect of metformin on the ovary or an epiphenomenon of the improved hormonal and metabolic pattern. Moreover, it is unclear, although very likely, if the ovulation itself could be a pivotal factor in the ovarian morphology changes. To this regard, further studies evaluating the intra-ovarian biochemical pattern in patients with different clinical response to metformin are guaranteed.

## Conclusion

Regardless of its systemic effects on hormonal and/or metabolic pattern, metformin administration modifies ovarian morphology in PCOS patients who ovulated under treatment probably by a direct peripheral action. However, further well-powered data are needed to completely explain the exact mechanisms by which metformin exerts its beneficial effects on the syndrome.

## Competing interests

The authors declare that they have no competing interests.
